# Response of *Caenorhabditis elegans* during subsequent infections with Gram positive and Gram negative bacteria

**DOI:** 10.1186/1471-2334-12-S1-P44

**Published:** 2012-05-04

**Authors:** Gnanasekaran Jeba Mercy, Krishnaswamy Balamurugan

**Affiliations:** 1 Department of Biotechnology, Alagappa University, Karaikudi-630 003, Tamil Nadu, India

## Background

The nematode *Caenorhabditis elegans* is one of the popular model hosts for the study of the evolutionarily conserved mechanism of microbial pathogenesis and innate immunity. *C. elegans* can be effectively used to study the dynamics of polymicrobial infections. *Proteus mirabilis*, an opportunistic pathogen, does not cause death in *C. elegans*. In this study the *C. elegans* was pre-infected with *Staphylococcus aureus* to make the *C. elegans* immunocompromised to study the effect of *P. mirabilis* in the host.

## Methods

This study involved in investigation of impact of subsequent infections at both physiological and molecular levels using *C. elegans* by killing assays and real time PCR analysis.

## Results

The study revealed that 12 h of *S. aureus* and 80 h of *P. mirabilis* subsequent infections reduced the life-span of 80% of the infected nematodes. Real time PCR analyses indicated the regulation of innate immune regulatory genes, lysozyme, CUB-like proteins, neuropeptide-like factors, transcription factors of MAP kinase and daf-2–daf-16, insulin-like signaling pathways and C-type lectin family members during polymicrobial infections, indicating possible role and contribution of the above players during host immune response against subsequent infections.

## Conclusions

Our findings demonstrate that the vulnerability of a host is an integral part of the *S. aureus* infection that enables the bacteria to subvert the host immune system, which can lead to the *P. mirabilis* to exert its pathogenicity in the host *C. elegans*.

